# Mutation Detection in Patients with Retinal Dystrophies Using Targeted Next Generation Sequencing

**DOI:** 10.1371/journal.pone.0145951

**Published:** 2016-01-14

**Authors:** Nicole Weisschuh, Anja K. Mayer, Tim M. Strom, Susanne Kohl, Nicola Glöckle, Max Schubach, Sten Andreasson, Antje Bernd, David G. Birch, Christian P. Hamel, John R. Heckenlively, Samuel G. Jacobson, Christina Kamme, Ulrich Kellner, Erdmute Kunstmann, Pietro Maffei, Charlotte M. Reiff, Klaus Rohrschneider, Thomas Rosenberg, Günther Rudolph, Rita Vámos, Balázs Varsányi, Richard G. Weleber, Bernd Wissinger

**Affiliations:** 1 Molecular Genetics Laboratory, Institute for Ophthalmic Research, Centre for Ophthalmology, University of Tuebingen, Tuebingen, Germany; 2 Institute of Human Genetics, Helmholtz Zentrum Muenchen, Neuherberg, Germany; 3 CeGaT GmbH, Tuebingen, Germany; 4 Institute of Medical Genetics and Human Genetics, Charité – Universitaetsmedizin Berlin, Berlin, Germany; 5 Department of Ophthalmology, Lund University, Lund, Sweden; 6 University Eye Hospital, Centre for Ophthalmology, University of Tuebingen, Tuebingen, Germany; 7 The Retina Foundation of the Southwest, Dallas, Texas, United States of America; 8 Genetic Sensory Diseases, CHU de Montpellier, Montpellier, France; 9 Department of Ophthalmology and Visual Sciences, Kellogg Eye Center, University of Michigan, Ann Arbor, Michigan, United States of America; 10 Scheie Eye Institute, Department of Ophthalmology, Perelman School of Medicine, University of Pennsylvania, Philadelphia, Pennsylvania, United States of America; 11 Rare Retinal Disease Center, AugenZentrum Siegburg, MVZ ADTC Siegburg GmbH, Siegburg, Germany; 12 Institute of Human Genetics, Julius-Maximilian-University, Wuerzburg, Germany; 13 Department of Medicine, University Hospital of Padua, Padua, Italy; 14 Eye Center, Albert-Ludwigs-University of Freiburg, Freiburg, Germany; 15 Department of Ophthalmology, University of Heidelberg, Heidelberg, Germany; 16 National Eye Clinic, Department of Ophthalmology, Glostrup Hospital, Glostrup, Denmark; 17 University Eye Hospital, Ludwig Maximilians University, Munich, Germany; 18 Department of Ophthalmology, Semmelweis University, Budapest, Hungary; 19 Department of Ophthalmology, University of Pécs Medical School, Pécs, Hungary; 20 Casey Eye Institute, Oregon Retinal Degeneration Center, Oregon Health & Science University, Portland, Oregon, United States of America; Innsbruck Medical University, AUSTRIA

## Abstract

Retinal dystrophies (RD) constitute a group of blinding diseases that are characterized by clinical variability and pronounced genetic heterogeneity. The different nonsyndromic and syndromic forms of RD can be attributed to mutations in more than 200 genes. Consequently, next generation sequencing (NGS) technologies are among the most promising approaches to identify mutations in RD. We screened a large cohort of patients comprising 89 independent cases and families with various subforms of RD applying different NGS platforms. While mutation screening in 50 cases was performed using a RD gene capture panel, 47 cases were analyzed using whole exome sequencing. One family was analyzed using whole genome sequencing. A detection rate of 61% was achieved including mutations in 34 known and two novel RD genes. A total of 69 distinct mutations were identified, including 39 novel mutations. Notably, genetic findings in several families were not consistent with the initial clinical diagnosis. Clinical reassessment resulted in refinement of the clinical diagnosis in some of these families and confirmed the broad clinical spectrum associated with mutations in RD genes.

## Introduction

Retinal dystrophies (RD) are among the disorders with the highest level of heterogeneity. This includes genetic heterogeneity, allelic heterogeneity as well as clinical heterogeneity. Molecular genetic studies in the last two decades revealed ~225 genes that are mutated in one or more of the various clinical subtypes of RD (https://sph.uth.edu/retnet/). Some of the clinical subtypes of RD can be caused by mutations in up to 60 different genes, e.g. in retinitis pigmentosa (RP). Adding to the genetic complexity there is considerable variation in clinical expression and overlap of symptoms of single disease entities, all of which may hamper making an exact clinical diagnosis. These obstacles have also practical implications for molecular diagnostics. Because it is difficult to predict the gene likely to be mutated, a gene-by-gene screening approach in RD patients is neither time- nor cost-efficient. On the other hand, establishing a molecular diagnosis is important for several reasons. It is vital for determining the recurrence risk for future children and therefore provides the basis for accurate genetic counseling. In many instances, it will also help to predict the clinical course, which is of central importance for the patients to plan and organize their professional and social lives. There is no effective cure for RD, however, ongoing clinical trials applying gene-replacement therapy approaches for several forms of RD have raised new hopes. Since these approaches require the identification of the causative mutation, the genetic diagnosis is an essential prerequisite.

The identification of the genetic defect in RD patients has been accelerated by the introduction of next-generation-sequencing technologies (NGS). Within the field of NGS platforms, the targeted capture of known disease genes (“disease panels”) has been proven superior in terms of coverage compared with whole exome sequencing (WES), especially for previous generations of exome capture reagents [1]. Exome capture kits of newer generations, however, show an improved performance and also offer the possibility to discover novel genes. On the other hand, neither conventional “disease panels” nor WES cover non-coding regions. Whole genome sequencing (WGS), besides its ability to sequence non-coding regions of the genome, has also been shown to outperform WES in the coding regions [1], but involves higher storage and analysis costs and is still challenging in terms of bioinformatic analysis.

In this study, we used a retinal capture panel, WES, and in one case WGS, to analyze—in a research context—89 unrelated cases with different forms of RD. Our results have important implications for the design and analysis strategy of routine genetic diagnostics in RD.

## Materials and Methods

### Clinical diagnosis and sample collection

The clinical diagnosis of RD was established by ophthalmological and/or electrophysiological examination in different clinical centers. The majority of patients were examined in the outpatient clinic for Inherited Retinal Dystrophies at the Centre for Ophthalmology, (Tuebingen, Germany). Others were clinically diagnosed at the outpatient clinic for Retinal Dystrophies at the University Eye Hospitals in Munich, Freiburg, and Berlin. Several cases were from Sweden, Denmark, France, Hungary and the USA. Genomic DNA of patients was extracted from peripheral blood using standard protocols. Samples from all patients and family members were recruited in accordance with the principles of the Declaration of Helsinki and were obtained with written informed consent accompanying the patients´ samples. The study was approved by the institutional review board of the Ethics Committee of the University Hospital of Tuebingen.

Except for a few cases most families had two or more affecteds. For the sake of brevity, in the following, we refer to multiple patients from one family as one case.

### Panel sequencing

We used a capture panel of 105 retinal disease genes (RD panel) to analyze 50 cases. Details of panel design, library preparation, capture sequencing and variant calling have already been published [2].

### Exome sequencing

**Fig 1:**
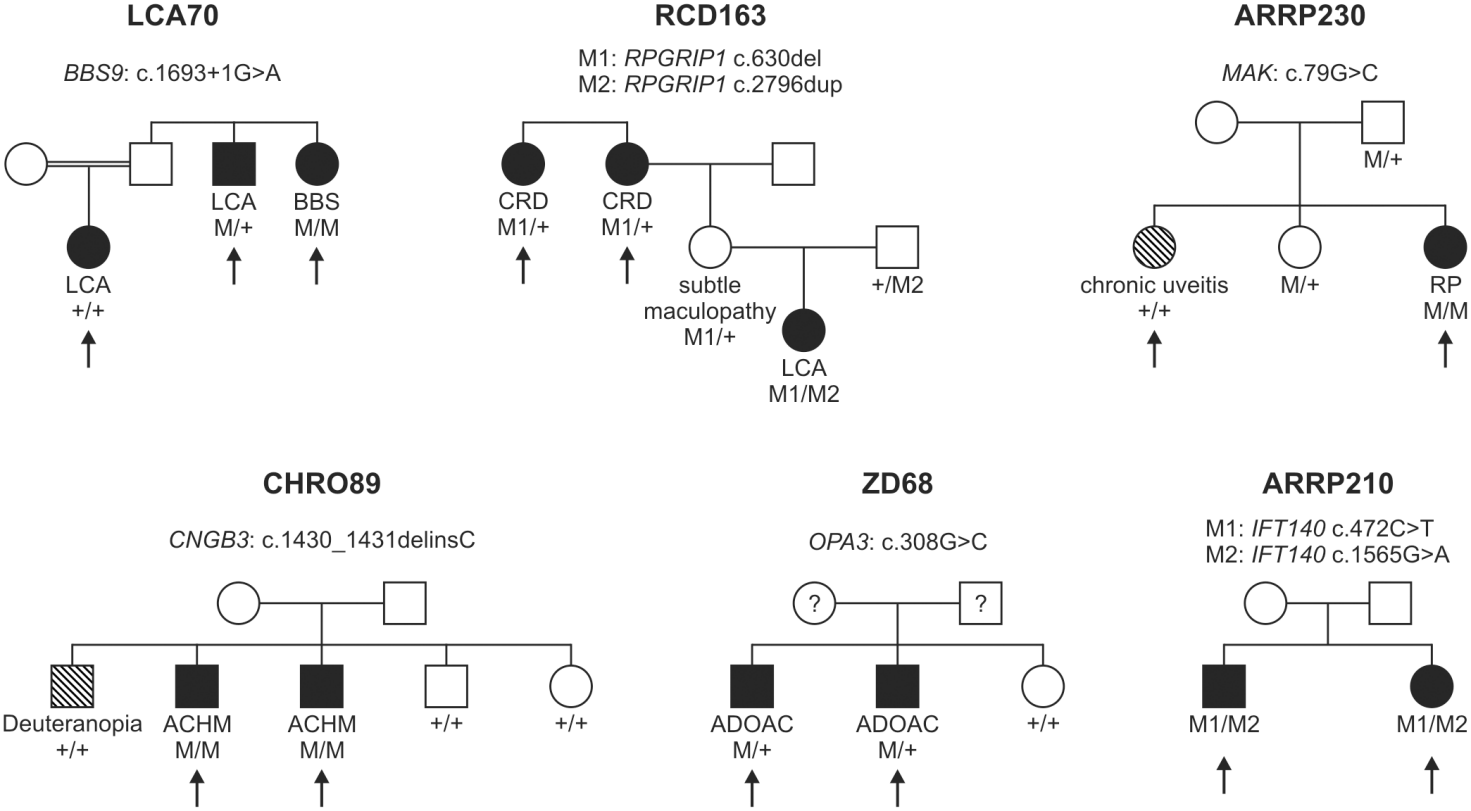
Pedigrees of six families discussed in detail in the manuscript. The arrows indicate the patients in whom NGS was performed. Family number and disease-causing mutation(s) are noted above each pedigree. The diagnosis of the patient and the genotype for each mutation are listed below each individual´s symbol. LCA, Leber congenital amaurosis; BBS, Bardet Biedl syndrome; CRD, cone-rod dystrophy; RP, retinitis pigmentosa; ACHM, achromatopsia; ADOAC, autosomal dominant optic atrophy and cataract.

We performed duo-based WES (two affected family members) in a cohort of 84 RD patients from 42 families, while in four cases only one exome was performed. For one family, WES was performed for three affected family members (pedigree LCA70 is depicted in Fig 1). Eight cases had been previously analyzed by our RD panel. Exomes were enriched using the SureSelect XT Human All Exon 50 Mb kit, versions 4 or 5 (Agilent Technologies, Santa Clara, CA, USA). Sequencing was performed on HiSeq 2500 systems (Illumina, San Diego, CA, USA). Reads were aligned against the human assembly hg19 (GRCh37) using Burrows—Wheeler Aligner version 0.7.5 [3]. We performed variant calling using SAMtools version 0.1.18 [4], PINDEL version 0.2.4t [5] and ExomeDepth version 1.0.0 [6]. Subsequently, variants were filtered using the SAMtools varFilter script and custom scripts. Shortly, only SNVs and indels in coding regions (nonsense, missense and canonical splice site variants as well as frameshift indels) having a potential effect on protein function *in silico* (assessed using predictions from PolyPhen-2 [http://genetics.bwh.harvard.edu/pph2/], SIFT [http://sift.bii.a-star.edu.sg/] and CADD [http://cadd.gs.washington.edu/]) were considered. From those, only private variants or those with a minor allele frequency <1% in a cohort of more than 66000 control individuals (ExAC Browser [http://exac.broadinstitute.org/]; and 6742 in-house exomes) were kept for subsequent analyses.

### Genome sequencing

One family was analyzed by whole genome sequencing. Details have already been published [7].

### Molecular validation of the candidate variants

All putative mutations identified by exome sequencing were validated using conventional Sanger sequencing according to the manufacturer´s protocols (3130XL Sequencer, Applied Biosystems, Weiterstadt, Germany) and tested for co-segregation within kinships.

Validation of the large deletion in the *PRPF31* gene was performed in the seven affected and eleven unaffected members of family ADRP32 using a long-distance PCR assay. To refine the breakpoint, we used a forward primer located in exon 3 (aagcaagccaaagcttcaga) and a reverse primer located in exon 14 (cctgtgggttcacaatctcc). For amplification, we applied a long distance PCR protocol using 80 ng of genomic DNA in a total volume of 25 μl containing 0.2 μM of each primer, 400 μM of each dNTP, LA Buffer (1X, without MgCl_2_), 0.5 mM MgCl_2_, and 2.5 U TaKaRa LA Taq DNA polymerase (Takara Bio Europe, Saint-Germain-en-Laye, France). Thermal cycling was performed with the following conditions: 1 min at 94°C followed by 14 cycles of 10 s at 98°C, 15 s at 55°C and 4 min at 68°C, a further 16 thermal cycles with an increment of 4 s/cycle for the elongation step, and a final add-on elongation for 10 min at 68°C.

Validation of the large deletion encompassing exons 15–22 of the *EYS* gene was performed using two duplex PCRs in the two affected and three unaffected members of family ARRP28. Due to the large intron sizes, breakpoints were not precisely defined. Briefly, primers were designed to co-amplify exons 14 and 15 in one PCR reaction, and exons 22 and 23 in another PCR reaction, respectively.

Screening for deep intronic variants (denoted as V1–V7) in the *ABCA4* gene [8] was performed in family CACD25 in which exome sequencing had revealed a single heterozygous missense mutation in *ABCA4*. Screening for V1–V7 was performed as described previously [9].

Characterization of the deep intronic mutation in *PROM1* in family RCD49 has been described before [7].

The mutational hot spot exon of *RPGR*, *ORF15*, was not accessible by our sequencing approaches in all cases due to its highly repetitive sequence. For the mutation screening of *ORF15* in unsolved RP families with absence of male-to-male transmission, we used the protocol described in Neidhardt et al. [10].

## Results and Discussion

### Mutation detection rate

The overall mutation detection rate of our study was 61% (54/89). More specifically, causative mutations were detected in 25 of 50 cases which were analyzed with our custom RD panel (50%). Average coverage was 750 reads per base pair with approximately 55% reads on target. In all cases but one, samples from additional family members were used to verify segregation of the sequence variants identified in the index patient.

Of the 25 cases that remained unsolved using our RD panel, eight were selected for subsequent analysis applying WES, in addition to a further 39 cases that were selected for direct WES analyses. Overall, 91 affected members from 47 families were subjected to WES. A total of 1017 Gigabases of data on target genomic regions were generated for the 91 samples with a mean coverage of the targeted region of 142 fold (minimum mean coverage was 89 fold). Following WES and subsequent analyses of intronic regions in two cases, we were able to identify pathogenic mutations in 29 cases (Table 1), thereby achieving a detection rate of 62% (29/47). In cases attributed to autosomal recessive inheritance that showed two heterozygous mutations, compound heterozygosity was confirmed by segregation analysis in all cases except three in which no DNA samples of additional family members were available. In cases attributed to autosomal dominant inheritance, co-segregation in two subsequent generations was confirmed in all cases except two owing to the lack of additional DNA samples. Of note, we did not observe any *de novo* mutations in our cohort.

**Table 1:** RD mutations identified in our cohort.

ID	Final diagnosis	Gene	Genotype	cDNA change	Protein change	Reference	Analysis
**Dominant inheritance**							
**ADRP1**	Retinitis pigmentosa	*RP1*	Heterozygous	c.2161del	p.G723Efs*15	[a]	WES
**ADRP18**	Retinitis pigmentosa	*NR2E3*	Heterozygous	c.166G>A	p.G56R	[11]	Panel
**ADRP32**	Retinitis pigmentosa	*PRPF31*	Heterozygous	Deletion exons 4–13	p.?	[12]	WES
**ADRP236**	Retinitis pigmentosa	*RP1*	Heterozygous	c.2329dup	p.R777Kfs*4	[a]	Panel
**ADRP298**	Retinitis pigmentosa	*RHO*	Heterozygous	c.874G>A	p.A292T	[a]	Panel
**ADRP308**	Retinitis pigmentosa	*RHO*	Heterozygous	c.512C>T	p.P171L	[13]	Panel
**BD121**	Exudative vitreoretinopathy	*FZD4*	Heterozygous	c.664A>G	p.W222R	[a]	WES
**CACD7**	Retinitis pigmentosa	*BEST1*	Heterozygous	c.584C>T	p.A195V	[14]	WES
**MB54**	Macular dystrophy	*GUCA1A*	Heterozygous	c.526C>T	p.L176F	[a]	WES
**MDS47**	Macular dystrophy	*BEST1*	Heterozygous	c.728C>T	p.A243V	[15]	WES
**MDS234**	Macular dystrophy	*RP1L1*	Heterozygous	c.133C>T	p.R45W	[16]	Panel
**RCD 70**	Cone-rod dystrophy	*CRX*	Heterozygous	c.502del	p.E168Sfs*19	[17]	Panel+WES
**RCD 82**	Cone-rod dystrophy	*PROM1*	Heterozygous	c.1117C>T	p.R373C	[18]	Panel+WES
**RCD512**	Cone-rod dystrophy	*PROM1*	Heterozygous	c.1117C>T	p.R373C	[18]	Panel
**ZD68**	Optic atrophy and cataract	*OPA3*	Heterozygous	c.308G>C	p.R103H	[a]	WES
**ZD218**	Macular dystrophy	*RP1L1*	Heterozygous	c.133C>T	p.R45W	[16]	Panel
**ZD302**	Macular dystrophy	*RP1L1*	Heterozygous	c.133C>T	p.R45W	[16]	Panel
**ZD367**	Cone dystrophy	*CRX*	Heterozygous	c.238G>A	p.E80K	[19]	Panel
**ZD396**	Cone-rod dystrophy	*GUCY2D*	Heterozygous	c.2513G>A	p.R838H	[20]	Panel
**Recessive inheritance**							
**ARRP17**	Retinitis pigmentosa	*CRB1*	Heterozygous	c.407G>A	p.C136Y	[a]	WES
			Heterozygous	c.1465G>T	p.E489*	[a]	
**ARRP28**	Retinitis pigmentosa	*EYS*	Homozygous	Deletion exons 15–22	p.?	[a]	Panel
**ARRP50**	Retinitis pigmentosa	*EYS*	Homozygous	c.5927+1G>T	p.?	[a]	Panel
**ARRP75**	Retinitis pigmentosa	*PDE6B*	Homozygous	c.1699C>T	p.Q567*	[21]	Panel
**ARRP82**	Retinitis pigmentosa	*USH2A*	Heterozygous	c.9433C>T	p.L3145F	[a]	WES
			Heterozygous	c.13335_13347del13ins4	p.E4445_S4449delinsDL	[a]	
**ARRP83**	Retinitis pigmentosa	*TULP1*	Homozygous	c.1604T>C	p.F535S	[21]	Panel
**ARRP138**	Retinitis pigmentosa	*CERKL*	Homozygous	c.1090C>T	p.R364*	[22]	Panel
**ARRP142**	Retinitis punctata albescens	*RLBP1*	Homozygous	c.398del	p.P133Qfs*126	[23]^§^	Panel
**ARRP165**	Retinitis pigmentosa	*IQCB1*	Homozygous	c.1558C>T	p.Q520*	[a]	Panel
**ARRP182**	Retinitis pigmentosa	*CLN3*	Homozygous	c.1213C>T	p.R405W	[24]	Panel+WES
**ARRP201**	Retinitis pigmentosa	*CYP4V2*	Heterozygous	c.283G>A	p.G95R	[25]	WES
			Heterozygous	c.1198C>T	p.R400C	[26]	
**ARRP210**	Retinitis pigmentosa	*IFT140*	Heterozygous	c.472C>T	p.R158W	[a]	WES
			Heterozygous	c.1565G>A	p.G522E	[27]	
**ARRP230**	Retinitis pigmentosa	*MAK*	Homozygous	c.79G>C	p.G27R	[28]	WES
**ARRP255**	Retinitis pigmentosa	*USH2A*	Heterozygous	c.2610C>A	p.C870*	[29]	WES
			Heterozygous	c.12261G>C	p.W4087C	[a]	
**CACD25**	Macular dystrophy	*ABCA4*	Heterozygous	c.5196+1137G>A^$^	p.?	[8]	WES
			Heterozygous	c.5311G>A	p.G1771R	[a]	
**CHRO89**	Achromatopsia	*CNGB3*	Homozygous	c.1430_1431delinsC	p.K477Tfs*17	[a]	Panel
**CHRO234**	Alström syndrome	*ALMS1*	Homozygous	c.1043G>A	p.W348*	[a]	WES
**CHRO249**	Cone-rod dystrophy	*RAB28*	Homozygous	c.565G>A	p.Q189*	[30]^§^	WES
**CHRO391**	Bardet Biedl syndrome	*BBS5*	Homozygous	c.790G>A	p.G264R	[a]	WES
**CHRO436**	Achromatopsia	*ATF6*	Heterozygous	c.797dup	p.N267*	[31]^§^	WES
			Heterozygous	c.1110dup	p.V371Sfs*3	[31]^§^	
**CHRO865**	Achromatopsia	*PDE6C*	Heterozygous	c.88_98del	p.V30Gfs*19	[a]	WES
			Heterozygous	c.1205T>A	p.V402E	[a]	
**LCA70**	Leber congenital amaurosis and Bardet Biedl syndrome	*BBS9*	Homozygous	c.1693+1G>A	p.?	[a]	WES
**MST177**	Stargardt disease	*ABCA4*	Heterozygous	c.2588G>C	p.G863A	[32]	WES
			Heterozygous	c.3898C>T	p.R1300*	[33]	
**RCD49**	Cone-rod dystrophy	*PROM1*	Homozygous	c.2077-521A>G	p.S684Ifs*21	[7]^§^	Panel+WES+WGS
**RCD69**	Cone-rod dystrophy	*CDHR1*	Heterozygous	c.1448A>G	p.E483G	[a]	Panel
			Heterozygous	c.2522_2528del	p.I841Sfs*119	[a]	
**RCD117**	Cone-rod dystrophy	*CERKL*	Heterozygous	c.356C>T	p.G119D	[a]	Panel
			Heterozygous	c.715G>A	p.R239*	[a]	
**RCD163**	Cone-rod dystrophy	*RPGRIP1*	Heterozygous	c.630del	p.H198Tfs*50	[a]	Panel
			Heterozygous	c.2796dup	p.E933*	[a]	
**RCD281**	Cone-rod dystrophy	*TULP1*	Heterozygous	c.1025G>A	p.R342Q	[34]	Panel
			Heterozygous	c.1496-6C>A	p.?	[35]	
**RCD285**	Cone-rod dystrophy	*PROM1*	Heterozygous	c.1327dup	p.S443Ffs*22	[a]	Panel+WES
			Heterozygous	c.1557C>A	p.Y519*	[36]	
**RCD500**	Leber congenital amaurosis	*CEP290*	Heterozygous	c.4723A>T	p.K1575*	[37]	Panel
			Heterozygous	c.5254C>T	p.R1752W	[21]	
**ZD345**	Cone dystrophy	*ABCA4*	Heterozygous	c.4139C>T	p.P1380L	[38]	Panel+WES
			Heterozygous	c.4253+4C>T	p.?	[39]	
**ZD410**	Cone dystrophy	*ABCA4*	Heterozygous	c.1622A>G	p.L541P	[40]	Panel
			Heterozygous	c.1643C>T	p.W548*	[a]	
**X-linked inheritance**							
**ADRP276**	Retinitis pigmentosa	*RPGR*	Hemizygous	c.1245+1G>T	p.?	[a]	Panel
**RCD291**	Cone-rod dystrophy	*RPGR*	Hemizygous	c.3011_3012del	p.E1004Gfs*74	[a]	WES
**Simplex cases**							
**LCA89**	Leber congenital amaurosis	*CEP290*	Heterozygous	c.3310-1_3310delinsAA	p.?	[a]	WES
			Heterozygous	c.5825A>C	p.Q1942P	[a]	

^§^Identified in this study but already published;

^$^not identified by WES but by subsequent screening for this variant;

a, this study.

A total of 69 distinct mutations were identified, including 39 novel mutations [11–40].

Although we counted them as being solved for the calculation of the total detection rate, three families of our cohort were only partially solved. All of them are now supposed to segregate two disease entities: two members of family LCA70 had a diagnosis of LCA while one sibling was diagnosed with Bardet Biedl syndrome (Fig 1). Applying WES to all three siblings we were able to identify a homozygous splice site mutation in *BBS9* that was unique for the patient with Bardet Biedl syndrome. The underlying mutation of the LCA phenotype in the remaining two siblings could not be identified so far.

One member of family RCD163 was diagnosed with LCA, while two siblings were diagnosed with cone-rod dystrophy (Fig 1). Using the RD panel, we could show that the patient with LCA was compound heterozygous for two frameshift mutations in *RPGRIP1* while the two other siblings were only heterozygous for one of the frameshift mutations. We excluded other variants in the coding regions and canonical splice site mutations of *RPGRIP1* in these patients using conventional Sanger sequencing. Whether they harbor a second disease-causing mutation in the non-coding regions of *RPGRIP1*, or whether their phenotype is caused by a second gene, remains unknown.

In family CHRO89, three brothers with a clinical diagnosis of achromatopsia/color vision deficiency were analyzed with the RD panel (Fig 1). Targeted sequencing revealed that only two of them harbour a homozygous frameshift mutation in the *CNGB3* gene while the third brother shows two wildtype alleles. The clinical difference between the brothers has already been noted in a prior clinical report [41]. Follow-up clinical examination showed that the non-segregating brother has reduced visual acuity and perifoveal depression of cone responses in the multifocal ERG, however, his color vision is not achromatic, but deuteranopic and he shows no nystagmus. The underlying mutation of his phenotype remains unclear.

Of note, eight cases that had been mutation-negative upon the analysis with our custom RD panel were selected for subsequent WES; five cases could be solved. Applying WES to family ARRP182 we were able to identify a known homozygous missense mutation in the *CLN3* gene. Yet at the time when the RD panel was applied in this case, it was not known that mutations in *CLN3* can cause nonsyndromic RP and therefore the gene was not included in the panel design. This clearly shows one of the major disadvantages of a panel-based sequencing approach: if a gene has not been linked to a specific disease at the time of its design, it will escape detection. In four cases, RCD70, RCD82, RCD285, and ZD345, WES detected mutations in *PROM1*, *CRX*, and *ABCA4*, respectively. Although these genes had been included in the RD panel design, the disease-causing variants in the four families were only detected by WES. Retrospective analysis of the panel sequencing data showed insufficient coverage of these regions. Later versions of the RD panel comprise additional probes and show a significantly improved coverage of these regions. Finally, family RCD49 could only be solved after applying WGS, simply due to the fact that the disease-causing mutation is located deep in an intron of *PROM1* and thereby could not be captured by the panel or by the WES approach.

### Comparison with other NGS studies on RD

In the present study, we applied a custom RD panel interrogating 105 RD genes to analyze 50 cases and were able to solve 25 cases. This detection rate of 50% is somewhat lower in comparison with a prior study using the same custom RD panel in a genetic diagnostic context setting (55% detection rate; [2]) as well as when compared with other studies which also used panel-based sequencing approaches. Eisenberger and colleagues [21] analyzed 55 genes in a cohort of 70 patients with RP and 56 patients with LCA and achieved an overall mutation detection rate of 70%. Other panel-based studies obtained similar results: in a cohort of 82 RP families from Northern Ireland disease-causing mutations were identified in 60% [42]. Another study analyzed 179 Chinese families with RD and achieved a detection rate of 55.3% [43]. The fact that we obtained only a detection rate of 50% in this study might be due to several reasons: 1) our cohort is more diverse concerning clinical phenotypes and inheritance traits; 2) we used a very early version of the RD panel which had some technical limitations; and 3) our cohort is somewhat biased since most cases had been extensively pre-screened for mutations in frequently affected genes applying Sanger sequencing and/or APEX arrays.

As for our detection rate of 62% in the cases that were analyzed by WES, a direct comparison with other studies is complicated due to differences in both cohort size and composition regarding clinical phenotypes and inheritance traits. Corton and colleagues used WES to analyze twelve Spanish families with presumed recessive RD and were able to solve ten of them [44]. Another study was able to identify disease-causing mutations in four of six Spanish families with an initial diagnosis of autosomal dominant RP [45]. A very recent study that analyzed 90 patients from 68 Israeli and Palestinian families with diagnoses of RP and LCA achieved a detection rate of 49% [46].

### Genetic heterogeneity

A total of 69 distinct mutations were identified in our study; 39 of them had not previously been reported (Tables 1 and 2). Among these novel mutations, 25 were nonsense, frameshift or splice site mutations presumably leading to functional null alleles while 14 were missense mutations that were predicted to have a deleterious effect on protein function *in silico*. Twelve of the novel missense mutations were absent from the ExAC database and two had a minor allele frequency of less than 0.00001.

**Table 2:** Classification of all identified putative pathogenic mutations.

	Novel	Previously reported
**Missense**	14	19
**Nonsense**	6	6
**Splice site**	3	2
**Small deletions/insertions**	14	1
**Large deletions**	1	1
**Deep intronic**	1	1
**Total**	39 (57%)	30 (43%)

With 36 genes implicated in disease in 54 families, and only few recurrent mutations in the same gene (Table 3), our observations reaffirm the known genetic heterogeneity of RD in an outbred European population. Similar genetic heterogeneity was also noted in 126 RP and LCA patients [21].

**Table 3:** Distribution of involved genes in our RD cohort.

Clinical diagnosis	Solved cases/total number of cases	Mutated genes (number of cases)
**ar Retinitis pigmentosa**	13/14	*CERKL* (1), *CLN3* (1), *CRB1* (1), *CYP4V2* (1), *EYS* (2), *IFT140* (1), *IQCB1* (1), *MAK* (1), *PDE6B* (1), *TULP1* (1), *USH2A* (2)
**ad Retinitis pigmentosa**	7/19	*BEST1* (1), *NR2E3* (1), *PRPF31* (1), *RHO* (2), *RP1* (2)
**X-linked Retinitis pigmentosa**	1/1	*RPGR* (1)
**ar Cone-rod dystrophy**	7/16	*CERKL* (1), *CDHR1* (1), *PROM1* (2), *RAB28* (1), *RPGRIP1* (1), *TULP1* (1)
**ad Cone-rod dystrophy**	4/5	*CRX* (1), *GUCY2D* (1), *PROM1* (2)
**X-linked Cone-rod dystrophy**	1/1	*RPGR* (1)
**ar Cone dystrophy**	2/6	*ABCA4* (2)
**ad Cone dystrophy**	1/2	*CRX* (1)
**ar Macular dystrophy**	1/2	*ABCA4* (1)
**ad Macular dystrophy**	5/6	*BEST1* (1), *GUCA1A* (1), *RP1L1* (3)
**Leber congenital amaurosis**	2/3	*CEP290* (2)
**Achromatopsia**	3/3	*ATF6* (1), *CNGB3* (1), *PDE6C* (1)
**Stargardt disease**	1/1	*ABCA4* (1)
**Alström syndrome**	1/1	*ALMS1* (1)
**Bardet Biedl syndrome**	2/2	*BBS5* (1), *BBS9* (1)*
**Optic atrophy and cataract**	1/1	*OPA3* (1)
**Exudative vitreoretinopathy**	1/1	*FZD4* (1)
**Retinitis punctata albescens**	1/1	*RLBP1* (1)
**Oligocone trichromacy**	0/2	-
**ad Vitreoretinochoroidopathy**	0/1	-
**Gyrate atrophy-like choroidal atrophy**	0/1	-

ar, autosomal recessive; ad, autosomal dominant;

*both Bardet Biedl syndrome and Leber congenital amaurosis are diagnosed in family LCA70.

Besides the identification of mutations in already known RD genes, WES led to the identification of two genes that had not previously been associated with RD, demonstrating a major advantage of WES in a research setting. The extreme genetic heterogeneity in RD usually makes it very unlikely to identify—in a limited study cohort—more than one family carrying mutations in a novel RD gene. However, such initial findings of potential candidates may find a match in databases listing single genetic findings (GeneMatcher; https://genematcher.org) or within the public domain of large consortia (e.g. the European Retinal Disease Consortium; http://www.erdc.info/) or will guide targeted screening in larger patient cohorts. Applying this strategy we were able to identify, or substantiate identification of, respectively, two novel genes associated with RD in our cohort of 47 families that underwent WES. In family CHRO249 we identified a homozygous nonsense mutation in *RAB28* that led to the first description of this gene being associated with cone-rod dystrophy [30] and in family CHRO436 we identified two heterozygous frameshift mutations in the *ATF6* gene, lending further support to our identification of *ATF6* as a novel gene for achromatopsia [31]. Replicates of initial findings may still be challenging for novel candidate genes of ultra-rare disease entities represented by several unsolved cases in our study cohort (e.g. autosomal dominant vitreoretinochoroidopathy in family BD49 and atrophy of the choroid and retina in family BD35, presenting with a fundus appearance of gyrate atrophy but without hyperornithinemia).

Two cases were shown to harbor pathogenic deep intronic mutations: family CACD25 is compound heterozygous for a missense mutation and a deep intronic mutation in *ABCA4* that affects splicing [8]. Two affected siblings of family RCD49 are homozygous for a deep intronic mutation in *PROM1* that leads to the activation of a cryptic exon [7].

### Revision of the initial clinical diagnosis

Our cohort included 47 cases attributed to autosomal recessive (53%) and 37 cases attributed to autosomal dominant inheritance (42%). Two cases were X-linked (2%) and three cases were isolated (3%). Final diagnoses of participating subjects included RP (34 cases), cone-rod dystrophy (23 cases), cone dystrophy (eight cases), macular dystrophy (eight cases), Leber congenital amaurosis (LCA; three cases), achromatopsia (three cases) and oligocone trichromacy (two cases). Additional single cases had final diagnoses of retinitis punctata albescens, autosomal dominant vitreoretinochoroidopathy, atrophy of the choroid and retina resembling gyrate atrophy but without hyperornithinemia, exudative vitreoretinopathy, optic atrophy, Alström syndrome, Bardet Biedl syndrome and Stargardt disease.

In several families we observed an inconsistency between expected findings based on the initial clinical diagnosis and the actual genetic result: family CHRO391, initially diagnosed with achromatopsia in childhood, was clinically re-examined since the only rare and potentially disease-causing exonic variants compatible with a model of autosomal recessive inheritance and shared by both siblings was a novel homozygous missense mutation in *BBS5*. Clinical re-examination revealed that the only symptom that could be attributed to Bardet Biedl syndrome is obesity. Neither polydactyly, renal dysfunction, hypogonadism, nor cognitive impairment was observed. However, the phenotype of Bardet Biedl syndrome is very variable and it has been shown that mutations in other *BBS* genes, like *BBS1* and *BBS2*, can cause mild forms or even nonsyndromic retinal dystrophy [47–48]. It is therefore likely, that the mutation in *BBS5* we found is the underlying cause of the phenotype in family CHRO391, especially since we did not find any variants in other genes that were considered pathogenic.

WES also helped to clarify distinct disease causes in family ARRP230: only one of two sisters initially diagnosed with RP was shown to be homozygous for a known missense mutation in *MAK* while the clinical symptoms of her sister, who does not carry the mutation, were shown to be due to chronic uveitis (Fig 1).

Family ZD68 had an initial diagnosis of cone dystrophy, and as a differential diagnosis optic atrophy and cataract (Fig 1). Genetic analysis showed that the two siblings harbor a novel missense mutation in the *OPA3* gene, which is implicated in autosomal dominant optic atrophy and cataract (ADOAC).

Two siblings of family ARRP210 were shown to harbor two heterozygous missense mutations in *IFT140* (Fig 1). This gene encodes a member of intraflagellar transport (IFT) proteins involved in bidirectional protein trafficking along the cilium. Mutations in genes coding for IFT components have been associated with several ciliopathies. In some instances, mutations might result in isolated forms of retinal degeneration, as has been shown for *IFT172* [49], and only recently for *IFT140* [50]. Prior to the latter publication, mutations in *IFT140* had only been described in patients with Mainzer-Saldino syndrome and Jeune asphyxiating thoracic dystrophy [27,51]. Both syndromes involve skeletal, renal, hepatic and retinal abnormalities. Extra-ocular symptoms are not apparent in the two siblings of family ARRP210 but could not yet be excluded by radiologic and internistic examinations. Nevertheless, our findings might confirm the recent finding that mutations in *IFT140* can result in nonsyndromic RP.

### Copy number variation

Copy number variations (CNV) are an important cause of human disease [52]. In RD, pathogenic CNVs have been described in a number of genes. For instance, deletions of one or more exons account for a considerable part of the mutation spectrum of *USH2A*, *EYS* and *PRPF31* (source: HGMD, http://www.biobase-international.com/product/hgmd). The accurate detection of large heterozygous deletions or duplications in WES data is considered one of the pitfalls of the method but is possible when applying suitable algorithms [53]. We used ExomeDepth [6] to discover CNVs in our data sets and were able to identify a large heterozygous deletion spanning exons 4–13 of the *PRPF31* gene in family ADRP32. In addition to the computational approach, we manually compared the number of exon reads for known RD genes in the unsolved cases but were not able to identify additional CNVs.

### RPGR ORF15

Despite the fact that our bioinformatic pipeline successfully identified a pathogenic deletion in *ORF15* of the *RPGR* gene in family RCD291, several issues prompted us to screen *ORF15* by conventional Sanger sequencing in unsolved RP families showing no male-to-male transmission: 1) The high incidence of X-linked RP among families initially classified as dominant [54], 2) the large proportion of *ORF15* mutations in X-linked RP [55], and 3) the poor coverage of *ORF15* in exome data due to its high repetitive nature. However, we were not able to identify additional disease-causing mutations in *ORF15* in our cohort.

### Unsolved cases—possible explanations

Thirty-five cases of our cohort could not be solved so far. Of these, 17 have only been analyzed by means of our custom RD panel. As discussed above, we used an early version of the RD panel which did not interrogate several more recently discovered RD-associated genes and also had some technical limitations. Of the 18 cases that remained unsolved after WES, seven cases were analyzed using a previous version of the exome capture kit. It has been shown that libraries obtained with the most recent Agilent V5 kit result in 94.57% of the targeted region covered by at least 20x compared with only 88.75% of the targeted region when the Agilent V4 kit was used [1]. Yet we did not achieve a higher detection rate in the group of cases that have been analyzed with V5 compared with those that have been analyzed with V4.

Of note, within the cohort that was analyzed by WES, we were able to solve a significantly lower fraction of cases with dominant inheritance (9/19) compared to cases with recessive inheritance (18/24). On average, our variant detection and annotation pipeline identified 100–150 sequence variants per family with dominant inheritance that were rare, potentially affecting protein function and shared by two affected family members. Even after filtering for retinal expression, several dozens of variants remained which made prioritization of novel candidate genes for dominantly inherited RD challenging.

Regardless of the inheritance pattern, it is likely that some causal variants will be structural or reside within non-coding regions. Genomic analyses for genes involved in retinal degeneration like *ABCA4* [8–9], *USH2A* [56–57] and *CEP290* [58] have shown that a probably underestimated number of patients harbor deep intronic variants that interfere with splicing. Some families might therefore be solved by WGS, like performed for family RCD49, in which we were able to identify a pathogenic deep intronic mutation in *PROM1*. However, computational analyses of WGS data sets are challenging and most likely only have prospect for success in families with multiple affecteds and evidence of linkage to known disease-gene loci.

### Lessons from our study

An important factor that might hamper identification of disease-causing mutations is inaccurate or insufficient pedigree information with regard to inheritance, disease entity or disease status. Although all our cases have been followed clinically for many years, we took into account the possibility of an imprecise clinical diagnosis or unexpected forms of inheritance since the wide phenotypic variability of RD with the clinical overlap of symptoms often hampers accurate clinical diagnosis. This obstacle was impressively demonstrated by the fact that our molecular findings led to the reclassification of the phenotype in several families. A good example for inaccurate pedigree information in our cohort is family ARRP230: both affected sisters had initially been diagnosed with retinitis pigmentosa but only one of them is homozygous for a known missense mutation in *MAK*. Retrospectively, the other sister was shown to suffer from chronic uveitis and not from retinitis pigmentosa. If we had only considered overlapping variants we would have missed the *MAK* variant. Moreover, three families in our cohort were shown to segregate two disease entities. This shows how important it is to analyze exome data sets with a hypothesis-free approach, especially in RD, with its pronounced clinical and genetic heterogeneity.

There is an increasing number of reports describing disease-causing mutations in non-coding sequences in RD families [8,58–61]. However, such reports are mainly based on incidental findings and there is a lack of a systematic study on the prevalence of such “cryptic” mutations. In our cohort of 89 unrelated cases, we were able to identify coding mutations in 52 cases while non-coding mutations were found in two cases, corresponding to 5% of previously unsolved cases; this confirms the necessity of analysis of regions outside of the coding exons. We therefore recommend that in future studies mutation screening should include at least as a second level screening, the analysis of non-coding regions of known RD disease genes.

In summary, our study confirms the diagnostic value of NGS platforms in the identification of mutations in a heterogeneous disease like RD. The advantage of WES to discover novel genes together with its reliable variant calling of coding regions and competitive prices, make it the technique of choice in the mutation screening of heterogeneous diseases.

## References

[1] Lelieveld SH, Spielmann M, Mundlos S, Veltman JA, Gilissen C. Comparison of Exome and Genome Sequencing Technologies for the Complete Capture of Protein-Coding Regions. Hum Mutat. 2015;36: 815–22. 10.1002/humu.22813 25973577 PMC4755152

[2] Glöckle N, Kohl S, Mohr J, Scheurenbrand T, Sprecher A, Weisschuh N et al Panel-based next generation sequencing as a reliable and efficient technique to detect mutations in unselected patients with retinal dystrophies. Eur J Hum Genet. 2014;22: 99–104. 10.1038/ejhg.2013.72 23591405 PMC3865404

[3] Li H, Durbin R. Fast and accurate short read alignment with Burrows-Wheeler transform. Bioinformatics. 2009;25: 1754–60. 10.1093/bioinformatics/btp324 19451168 PMC2705234

[4] Li H, Handsaker B, Wysoker A, Fennell T, Ruan J, Homer N et al The Sequence Alignment/Map format and SAMtools. Bioinformatics. 2009;25: 2078–9. 10.1093/bioinformatics/btp352 19505943 PMC2723002

[5] Ye K, Schulz MH, Long Q, Apweiler R, Ning Z. Pindel: a pattern growth approach to detect break points of large deletions and medium sized insertions from paired-end short reads. Bioinformatics. 2009;25: 2865–71. 10.1093/bioinformatics/btp394 19561018 PMC2781750

[6] Plagnol V, Curtis J, Epstein M, Mok KY, Stebbings E, Grigoriadou S et al A robust model for read count data in exome sequencing experiments and implications for copy number variant calling. Bioinformatics. 2012;28: 2747–54. 10.1093/bioinformatics/bts526 22942019 PMC3476336

[7] Mayer AK, Rohrschneider K, Strom TM, Glöckle N, Kohl S, Wissinger B et al Homozygosity mapping and whole-genome sequencing reveals a deep intronic PROM1 mutation causing cone-rod dystrophy by pseudoexon activation. Eur J Hum Genet. 2015 7 8. [Epub ahead of print] 10.1038/ejhg.2015.144 PMC4755378 26153215

[8] Braun TA, Mullins RF, Wagner AH, Andorf JL, Johnston RM, Bakall BB et al Non-exomic and synonymous variants in ABCA4 are an important cause of Stargardt disease. Hum Mol Genet. 2013;22: 5136–45. 10.1093/hmg/ddt367 23918662 PMC3842174

[9] Bauwens M, De Zaeytijd J, Weisschuh N, Kohl S, Meire F, Dahan K et al An augmented ABCA4 screen targeting noncoding regions reveals a deep intronic founder variant in Belgian Stargardt patients. Hum Mutat. 2015;36: 39–42. 10.1002/humu.22716 25346251

[10] Neidhardt J, Glaus E, Lorenz B, Netzer C, Li Y, Schambeck M et al Identification of novel mutations in X-linked retinitis pigmentosa families and implications for diagnostic testing. Mol Vis. 2008;14: 1081–93. 18552978 PMC2426717

[11] Coppieters F, Leroy BP, Beysen D, Hellemans J, De Bosscher K, Haegeman G et al Recurrent mutation in the first zinc finger of the orphan nuclear receptor NR2E3 causes autosomal dominant retinitis pigmentosa. Am J Hum Genet. 2007;81: 147–57. 17564971 10.1086/518426 PMC1950922

[12] Sullivan LS, Bowne SJ, Seaman CR, Blanton SH, Lewis RA, Heckenlively JR et al Genomic rearrangements of the PRPF31 gene account for 2.5% of autosomal dominant retinitis pigmentosa. Invest Ophthalmol Vis Sci. 2006;47: 4579–88. 17003455 10.1167/iovs.06-0440 PMC2778205

[13] Dryja TP, Hahn LB, Cowley GS, McGee TL, Berson EL. Mutation spectrum of the rhodopsin gene among patients with autosomal dominant retinitis pigmentosa. Proc Natl Acad Sci U S A. 1991;88: 9370–4. 1833777 10.1073/pnas.88.20.9370 PMC52716

[14] Lotery AJ, Munier FL, Fishman GA, Weleber RG, Jacobson SG, Affatigato LM et al Allelic variation in the VMD2 gene in best disease and age-related macular degeneration. Invest Ophthalmol Vis Sci. 2000;41: 1291–6. 10798642

[15] White K, Marquardt A, Weber BH. VMD2 mutations in vitelliform macular dystrophy (Best disease) and other maculopathies. Hum Mutat. 2000;15: 301–8. 10737974 10.1002/(SICI)1098-1004(200004)15:4<301::AID-HUMU1>3.0.CO;2-N

[16] Akahori M, Tsunoda K, Miyake Y, Fukuda Y, Ishiura H, Tsuji S et al Dominant mutations in RP1L1 are responsible for occult macular dystrophy. Am J Hum Genet. 2010;87: 424–9. 10.1016/j.ajhg.2010.08.009 20826268 PMC2933341

[17] Freund CL, Gregory-Evans CY, Furukawa T, Papaioannou M, Looser J, Ploder L et al Cone-rod dystrophy due to mutations in a novel photoreceptor-specific homeobox gene (CRX) essential for maintenance of the photoreceptor. Cell. 1997;91: 543–53. 9390563 10.1016/s0092-8674(00)80440-7

[18] Yang Z, Chen Y, Lillo C, Chien J, Yu Z, Michaelides M et al Mutant prominin 1 found in patients with macular degeneration disrupts photoreceptor disk morphogenesis in mice. J Clin Invest. 2008;118: 2908–16. 10.1172/JCI35891 18654668 PMC2483685

[19] Sankila EM, Joensuu TH, Hämäläinen RH, Raitanen N, Valle O, Ignatius J et al A CRX mutation in a Finnish family with dominant cone-rod retinal dystrophy. Hum Mutat. 2000;16: 94. 10.1002/1098-1004(200007)16:1<94::AID-HUMU25>3.0.CO;2-T 10874321

[20] Payne AM, Morris AG, Downes SM, Johnson S, Bird AC, Moore AT et al Clustering and frequency of mutations in the retinal guanylate cyclase (GUCY2D) gene in patients with dominant cone-rod dystrophies. J Med Genet. 2001;38: 611–4. 11565546 10.1136/jmg.38.9.611 PMC1734946

[21] Eisenberger T, Neuhaus C, Khan AO, Decker C, Preising MN, Friedburg C et al Increasing the yield in targeted next-generation sequencing by implicating CNV analysis, non-coding exons and the overall variant load: the example of retinal dystrophies. PLoS One. 2013;8: e78496 10.1371/journal.pone.0078496 24265693 PMC3827063

[22] Boulanger-Scemama E, El Shamieh S, Démontant V, Condroyer C, Antonio A, Michiels C et al Next-generation sequencing applied to a large French cone and cone-rod dystrophy cohort: mutation spectrum and new genotype-phenotype correlation. Orphanet J Rare Dis. 2015;10: 85 10.1186/s13023-015-0300-3 26103963 PMC4566196

[23] Hipp S, Zobor G, Glöckle N, Mohr J, Kohl S, Zrenner E et al Phenotype variations of retinal dystrophies caused by mutations in the RLBP1 gene. Acta Ophthalmol. 2015;93: e281–6. 10.1111/aos.12573 25429852

[24] Wang F, Wang H, Tuan HF, Nguyen DH, Sun V, Keser V et al Next generation sequencing-based molecular diagnosis of retinitis pigmentosa: identification of a novel genotype-phenotype correlation and clinical refinements. Hum Genet. 2014;133: 331–45. 10.1007/s00439-013-1381-5 24154662 PMC3945441

[25] Shan M, Dong B, Zhao X, Wang J, Li G, Yang Y et al Novel mutations in the CYP4V2 gene associated with Bietti crystalline corneoretinal dystrophy. Mol Vis. 2005;11: 738–43. 16179904

[26] Lai TY, Ng TK, Tam PO, Yam GH, Ngai JW, Chan WM et al Genotype phenotype analysis of Bietti's crystalline dystrophy in patients with CYP4V2 mutations. Invest Ophthalmol Vis Sci. 2007;48: 5212–20. 17962476 10.1167/iovs.07-0660

[27] Perrault I, Saunier S, Hanein S, Filhol E, Bizet AA, Collins F et al Mainzer-Saldino syndrome is a ciliopathy caused by IFT140 mutations. Am J Hum Genet. 2012;90: 864–70. 10.1016/j.ajhg.2012.03.006 22503633 PMC3376548

[28] Ozgül RK, Siemiatkowska AM, Yücel D, Myers CA, Collin RW, Zonneveld MN et al Exome sequencing and cis-regulatory mapping identify mutations in MAK, a gene encoding a regulator of ciliary length, as a cause of retinitis pigmentosa. Am J Hum Genet. 2011;89: 253–64. 10.1016/j.ajhg.2011.07.005 21835304 PMC3155188

[29] Le Quesne Stabej P, Saihan Z, Rangesh N, Steele-Stallard HB, Ambrose J, Coffey A et al Comprehensive sequence analysis of nine Usher syndrome genes in the UK National Collaborative Usher Study. J Med Genet. 2012;49: 27–36. 10.1136/jmedgenet-2011-100468 22135276 PMC3678402

[30] Roosing S, Rohrschneider K, Beryozkin A, Sharon D, Weisschuh N, Staller J et al Mutations in RAB28, encoding a farnesylated small GTPase, are associated with autosomal-recessive cone-rod dystrophy. Am J Hum Genet. 2013;93: 110–7. 10.1016/j.ajhg.2013.05.005 23746546 PMC3710761

[31] Kohl S, Zobor D, Chiang WC, Weisschuh N, Staller J, Menendez IG et al Mutations in the unfolded protein response regulator ATF6 cause the cone dysfunction disorder achromatopsia. Nat Genet. 2015;47: 757–65. 10.1038/ng.3319 26029869 PMC4610820

[32] Allikmets R, Singh N, Sun H, Shroyer NF, Hutchinson A, Chidambaram A et al A photoreceptor cell-specific ATP-binding transporter gene (ABCR) is mutated in recessive Stargardt macular dystrophy. Nat Genet. 1997;15: 236–46. 9054934 10.1038/ng0397-236

[33] Rivera A, White K, Stöhr H, Steiner K, Hemmrich N, Grimm T et al A comprehensive survey of sequence variation in the ABCA4 (ABCR) gene in Stargardt disease and age-related macular degeneration. Am J Hum Genet. 2000;67: 800–13. 10958763 10.1086/303090 PMC1287885

[34] Hebrard M, Manes G, Bocquet B, Meunier I, Coustes-Chazalette D, Hérald E et al Combining gene mapping and phenotype assessment for fast mutation finding in non-consanguineous autosomal recessive retinitis pigmentosa families. Eur J Hum Genet. 2011;19: 1256–63. 10.1038/ejhg.2011.133 21792230 PMC3230368

[35] Gu S, Lennon A, Li Y, Lorenz B, Fossarello M, North M et al Tubby-like protein-1 mutations in autosomal recessive retinitis pigmentosa. Lancet. 1998;351: 1103–4. 9660588 10.1016/S0140-6736(05)79384-3

[36] Song J, Smaoui N, Ayyagari R, Stiles D, Benhamed S, MacDonald IM et al High-throughput retina-array for screening 93 genes involved in inherited retinal dystrophy. Invest Ophthalmol Vis Sci. 2011;52: 9053–60. 10.1167/iovs.11-7978 22025579 PMC3231844

[37] Perrault I, Delphin N, Hanein S, Gerber S, Dufier JL, Roche O et al Spectrum of NPHP6/CEP290 mutations in Leber congenital amaurosis and delineation of the associated phenotype. Hum Mutat. 2007;28: 416. 10.1002/humu.9485 17345604

[38] Lewis RA, Shroyer NF, Singh N, Allikmets R, Hutchinson A, Li Y et al Genotype/Phenotype analysis of a photoreceptor-specific ATP-binding cassette transporter gene, ABCR, in Stargardt disease. Am J Hum Genet. 1999;64: 422–34. 9973280 10.1086/302251 PMC1377752

[39] Ozgül RK, Durukan H, Turan A, Oner C, Ogüs A, Farber DB. Molecular analysis of the ABCA4 gene in Turkish patients with Stargardt disease and retinitis pigmentosa. Hum Mutat. 2004;23: 523. 10.1002/humu.9236 15108289

[40] Rozet JM, Gerber S, Souied E, Perrault I, Châtelin S, Ghazi I et al Spectrum of ABCR gene mutations in autosomal recessive macular dystrophies. Eur J Hum Genet. 1998;6: 291–5. 9781034 10.1038/sj.ejhg.5200221

[41] Jaeger W, Krastel H. Complete and incomplete congenital achromatopsia in one sibship In: Huber A, Klein E, editors. Proc Congr Neurogenetics and Neuroophthalmology, Zürich 1981 pp.241–245.

[42] Zhao L, Wang F, Wang H, Li Y, Alexander S, Wang K et al Next-generation sequencing-based molecular diagnosis of 82 retinitis pigmentosa probands from Northern Ireland. Hum Genet. 2015;134: 217–30. 10.1007/s00439-014-1512-7 25472526 PMC4347882

[43] Huang XF, Huang F, Wu KC, Wu J, Chen J, Pang CP et al Genotype-phenotype correlation and mutation spectrum in a large cohort of patients with inherited retinal dystrophy revealed by next-generation sequencing. Genet Med. 2015;17: 271–8. 10.1038/gim.2014.138 25356976

[44] Corton M, Nishiguchi KM, Avila-Fernández A, Nikopoulos K, Riveiro-Alvarez R, Tatu SD et al Exome sequencing of index patients with retinal dystrophies as a tool for molecular diagnosis. PLoS One. 2013;8: e65574 10.1371/journal.pone.0065574 23940504 PMC3683009

[45] Almoguera B, Li J, Fernandez-San Jose P, Liu Y, March M, Pellegrino R et al Application of Whole Exome Sequencing in Six Families with an Initial Diagnosis of Autosomal Dominant Retinitis Pigmentosa: Lessons Learned. PLoS One. 2015;10: e0133624 10.1371/journal.pone.0133624 26197217 PMC4509755

[46] Beryozkin A, Shevah E, Kimchi A, Mizrahi-Meissonnier L, Khateb S, Ratnapriya R et al Whole Exome Sequencing Reveals Mutations in Known Retinal Disease Genes in 33 out of 68 Israeli Families with Inherited Retinopathies. Sci Rep. 2015;5: 13187 10.1038/srep13187 26306921 PMC4549705

[47] Estrada-Cuzcano A, Koenekoop RK, Senechal A, De Baere EB, de Ravel T, Banfi S et al BBS1 mutations in a wide spectrum of phenotypes ranging from nonsyndromic retinitis pigmentosa to Bardet-Biedl syndrome. Arch Ophthalmol. 2012;130: 1425–32. 10.1001/archophthalmol.2012.2434 23143442

[48] Shevach E, Ali M, Mizrahi-Meissonnier L, McKibbin M, El-Asrag M, Watson CM et al Association between missense mutations in the BBS2 gene and nonsyndromic retinitis pigmentosa. JAMA Ophthalmol. 2015;133: 312–8. 10.1001/jamaophthalmol.2014.5251 25541840

[49] Bujakowska KM, Zhang Q, Siemiatkowska AM, Liu Q, Place E, Falk MJ et al Mutations in IFT172 cause isolated retinal degeneration and Bardet-Biedl syndrome. Hum Mol Genet. 2015;24: 230–42. 10.1093/hmg/ddu441 25168386 PMC4326328

[50] Xu M, Yang L, Wang F, Li H, Wang X, Wang W et al Mutations in human IFT140 cause non-syndromic retinal degeneration. Hum Genet. 2015 7 28. [Epub ahead of print] 10.1007/s00439-015-1586-x PMC4565766 26216056

[51] Schmidts M, Frank V, Eisenberger T, Al Turki S, Bizet AA, Antony D et al Combined NGS approaches identify mutations in the intraflagellar transport gene IFT140 in skeletal ciliopathies with early progressive kidney Disease. Hum Mutat. 2013;34: 714–24. 10.1002/humu.22294 23418020 PMC4226634

[52] Almal SH, Padh H. Implications of gene copy-number variation in health and diseases. J Hum Genet. 2012;57: 6–13. 10.1038/jhg.2011.108 21956041

[53] Tan R, Wang Y, Kleinstein SE, Liu Y, Zhu X, Guo H et al An evaluation of copy number variation detection tools from whole-exome sequencing data. Hum Mutat. 2014;35: 899–907. 10.1002/humu.22537 24599517

[54] Churchill JD, Bowne SJ, Sullivan LS, Lewis RA, Wheaton DK, Birch DG et al Mutations in the X-linked retinitis pigmentosa genes RPGR and RP2 found in 8.5% of families with a provisional diagnosis of autosomal dominant retinitis pigmentosa. Invest Ophthalmol Vis Sci. 2013;54: 1411–6. 10.1167/iovs.12-11541 23372056 PMC3597192

[55] Vervoort R, Lennon A, Bird AC, Tulloch B, Axton R, Miano MG et al Mutational hot spot within a new RPGR exon in X-linked retinitis pigmentosa. Nat Genet. 2000;25: 462–6. 10932196 10.1038/78182

[56] Vaché C, Besnard T, le Berre P, García-García G, Baux D, Larrieu L et al Usher syndrome type 2 caused by activation of an USH2A pseudoexon: implications for diagnosis and therapy. Hum Mutat. 2012;33: 104–8. 10.1002/humu.21634 22009552

[57] Steele-Stallard HB, Le Quesne Stabej P, Lenassi E, Luxon LM, Claustres M, Roux AF et al Screening for duplications, deletions and a common intronic mutation detects 35% of second mutations in patients with USH2A monoallelic mutations on Sanger sequencing. Orphanet J Rare Dis. 2013;8: 122 10.1186/1750-1172-8-122 23924366 PMC3751126

[58] den Hollander AI, Koenekoop RK, Yzer S, Lopez I, Arends ML, Voesenek KE et al Mutations in the CEP290 (NPHP6) gene are a frequent cause of Leber congenital amaurosis. Am J Hum Genet. 2006;79: 556–61. 16909394 10.1086/507318 PMC1559533

[59] Rio Frio T, McGee TL, Wade NM, Iseli C, Beckmann JS, Berson EL et al A single-base substitution within an intronic repetitive element causes dominant retinitis pigmentosa with reduced penetrance. Hum Mutat. 2009;30: 1340–7. 10.1002/humu.21071 19618371 PMC2753193

[60] Marshall JD, Muller J, Collin GB, Milan G, Kingsmore SF, Dinwiddie D et al Alström Syndrome: Mutation Spectrum of ALMS1. Hum Mutat. 2015;36: 660–8. 10.1002/humu.22796 25846608 PMC4475486

[61] Naruto T, Okamoto N, Masuda K, Endo T, Hatsukawa Y, Kohmoto T et al Deep intronic GPR143 mutation in a Japanese family with ocular albinism. Sci Rep. 2015;5: 11334 10.1038/srep11334 26061757 PMC4650666

